# Cold atmospheric plasma treatment induces oxidative stress and alters microbial community profile in the leaves of sweet basil (*Ocimum basilicum* var. Kiera) plant

**DOI:** 10.1111/1750-3841.70066

**Published:** 2025-02-14

**Authors:** Andrea R. Gilbert‐Eckman, Mairui Gao, Ryan A. Blaustein, Rohan V. Tikekar

**Affiliations:** ^1^ Department of Nutrition and Food Science University of Maryland College Park Maryland USA

**Keywords:** basil, cold atmosphere plasma, plant microbiome, reactive oxygen species

## Abstract

**Abstract:**

The oxidative species generated by cold atmospheric plasma (CAP) treatment can impact the plant stress response system. We hypothesized that this response is not limited to the site of CAP application and it is transmitted through the plant. The resulting stress response can influence the plant microbiome on the intact plant. These hypotheses were tested by the application of CAP to live sweet basil (*Ocimum basilicum* var. Kiera). A single upper leaf of the plant underwent a 60 s CAP treatment at three different wattage intensity levels. Reactive oxygen species (ROS) generation in directly treated leaves and leaves in the vicinity of the treatment site (i.e., one, two, or three nodes away) was measured using the fluorescein degradation assay (ex/em: 485/525). Leaves directly exposed to CAP showed a marked increase in ROS production. Interestingly, basil leaves not directly treated by CAP also showed a significant (*p* < 0.05) increase in ROS generation compared to untreated control, extending to the two nearest nodes from the treatment site in all plants tested. The leaf microbiomes were evaluated using 16S rRNA gene sequencing. CAP appeared to drive restructuring of the leaf microbiota profiles, despite maintaining a similar α‐diversity. CAP treatment intensity led to significant differences (*p* < 0.05) in the relative abundances of a variety of dominant bacterial families (e.g., Psuedomonadaceae and Streptomycetaceae) and phyla, and the effects on certain taxa were dependent on leaf distance from the treatment site. CAP's ability to restructure plant microbiota may have applications to improve produce microbial safety and shelf‐life.

**Practical Application:**

Cold atmospheric plasma induces a stress response in a living plant beyond the site of application. This response includes an increase in the production of reactive oxygen species that can trigger pathways to enhance the production of phytochemicals. CAP treatment also alters the microbial community profile, possibly through plant stress response. Results from this study can be useful in developing CAP treatment of intact plant for improved growth, production of health‐benefiting phytochemicals, and managing its microbiota.

## 1 INTRODUCTION

Cold atmospheric plasma (CAP) is a nonthermal processing technology with increasingly recognized potential in the food industry, including fresh produce. There are many methods to produce plasmas at atmospheric temperature and pressure; however, plasmas produced from difficult‐to‐ionize gases such as air and oxygen have stronger antimicrobial applications than that produced from inert gases such as helium or argon (Conrads & Schmidt, 2000; Luan et al., 2019; Niemira, 2012a, 2012b). Although there are a variety of electrode types that can be used to generate plasma, the dielectric barrier discharge (DBD) plasma devices are well suited for use on leafy greens. In this configuration, the DBD electrode serves as the charged electrode, whereas the substrate food serves as the ground electrode. When a high‐voltage current runs through the charged electrode, plasma species such as reactive oxygen and nitrogen species and ultraviolet (UV) light are generated in the interelectrode space (Domonkos et al., 2021; Laroussi, 2002; Laroussi et al., 2003, 2002). The plasma species inactivate microbes via three main routes, the chemical interactions of radicals, reactive species, and charged particles with cell membranes, damage to membranes and organelles due to UV radiation, and UV DNA damage; however, UV is not the main driver of microbial inactivation (Dobrynin et al., 2009; Laroussi et al., 2002; Luan et al., 2019; Niemira, 2012a, 2012b). DBD plasma treatment does not require a charge gas as the surrounding air is sufficient for generating antimicrobial gaseous species (Domonkos et al., 2021; Luan et al., 2019; Niemira, 2012a). This method also eliminates the need for water in the treatment (Gil et al., 2009, 2015; Luan et al., 2019; Niemira, 2012b), and unlike UV and other light‐based sanitization systems, “line of sight” or direct contact is not required for effective treatment (Herrmann et al., 1999; Niemira, 2012a). Therefore, CAP has been extensively studied for fresh produce‐related applications as discussed in several comprehensive review publications (Mao et al., 2021; Pan et al., 2019). However, interactions of CAP with the live plant leaf tissues and its implications on the plant stress and its phyllosphere are much less studied.

Fresh fruits and vegetables, like all plants, naturally harbor a diverse microbiota that plays a role in plant health and development. The phyllosphere, including the edible components of fresh produce, contains microorganisms derived from soil, seeds, and air at different stages of plant growth (De Mandal & Jeon, 2023; Sohrabi et al., 2023). For example, leafy green vegetables typically contain 10^2^–10^8^ bacteria CFU/g (Hummerick et al., 2021; Korir et al., 2016), which may be represented by hundreds or even thousands of different bacterial genera (Artimová et al., 2023). Although CAP treatment for food safety applications aims to reduce overall microbial loads to lower risks for contamination by pathogens and potential spoilage agents, the posttreatment plant‐associated microbiota may remain diverse, albeit altered, even after responding to the introduced selection pressures (Gu et al., 2020; Rosberg et al., 2021). Considering the increasingly recognized importance of microbial exposures and probiotics in supporting the human microbiome, food processing technologies that maintain key aspects of edible plant microbiome diversity (i.e., not including pathogenic taxa) may be favorable for consumers (Soto‐Giron et al., 2021). Understanding how CAP restructures microbial diversity on fresh produce remains unknown.

Abiotic stresses such as UV light and oxidizers are known to generate stress response within the live plants, including oxidative stress, that is, transmitted through the entire plant. This stress response frequently includes upregulation in shikimate, phenylpropanoid, and flavonoid synthesis pathways. These pathways are known to produce diverse secondary metabolites such as anthocyanins, flavonols and catechins, caffeic acid, ferulic acid conjugates, gallic acid, and epigallocatechin, which help the plant combat stress (Kumar et al., 2023). Thus, plant experiencing oxidative stress is usually an early sign that these pathways may be upregulated (Solecka, 1997; Tripathy & Oelmuller, 2012). As CAP is composed of reactive oxidative species and UV light, it led us to two research questions: Does CAP trigger an oxidative stress within the live plant? Is this stress limited to the CAP application site or propagated through the plant? Additionally, as plant stress response is also known to have interactions with the microorganisms that make up the phyllosphere (Ali & Baek, 2020; Omae & Tsuda, 2022), it is reasonable to expect that CAP treatments may have indirect and lasting impacts on the commensal plant microbiome. These impacts will likely also be experienced by fresh produce in general as plant stress responses persist in tissue where metabolic activity has not been stopped by processing. We tested these two hypotheses by evaluating the systemic oxidative stress response of CAP in sweet basil (*Ocimum basilicum*) plant and changes in its resident microflora following the CAP treatment. Sweet basil was chosen for this experiment as it is the most popular fresh and living herb (live plant sold as fresh produce in a grocery store) sold in the United States.

## 2 MATERIALS AND METHODS

### 2.1 Fresh produce

Sweet basil (var. Kiera) plants were also purchased from a local grocery store and stored in a portable growth chamber on a 12‐h light cycle until treatment (ViaGrow, Atlanta, GA).

### 2.2 Plasma device

**FIGURE 1:**
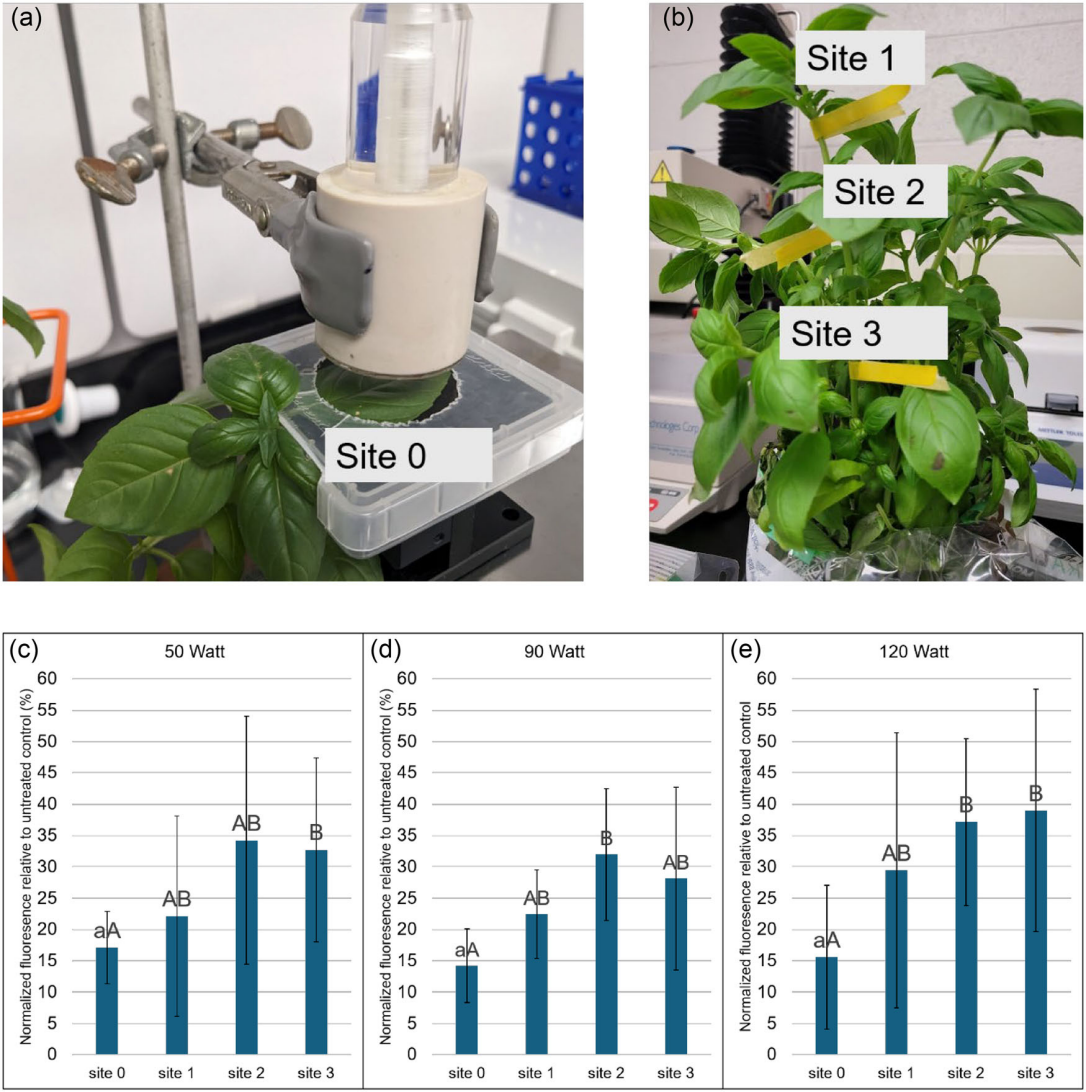
(a) Set up of live basil plant for dielectric barrier discharge (DBD) plasma treatment of Site 0. (b) Location of Sites 1–3 on basil plant. (c–e) Reactive oxygen species (ROS) generation in sweet basil plants at the site of application (Site 0) and progressively distant sites (Sites 1–3) as measured by relative loss of fluorescence from fluorescein with respect to untreated control upon exposure to (c) 50 W, (d) 90 W, and (e) 120 W DBD‐cold atmospheric plasma (CAP) treatment for 60 s. In subparts (c–e): different uppercase letters indicate significant differences at *p* ≤ 0.05 within the treatment wattage and different lowercase letters indicate significant difference between three Site 0 treatment sites at *p* < 0.05. Statistical comparison between Site 1–3 and at different wattage levels was not performed.

A benchtop 30 kV plasma device was purchased from Advanced Plasma Solutions, and this power source and DBD electrodes from the supplier were used for all experiments. All experiments with the plasma device were carried out in a fume hood as a precaution against excess ozone exposure or accidental excessive electrical arcing. Plant samples were treated with the DBD electrode after being placed on an adjustable stage and clamped in place using a magenta box lid (PlantMedia) that had been cut to allow the charged electrode to interact with the substrate (Figure 1).

When samples were treated, the fine adjustment of the stand was used to ensure that the treated sample was between 5 and 2.5 mm from the ground electrode, and samples were not completely flattened for treatment to prevent physical injury to the plant tissue from contributing to the stress response readings. The samples were treated at three different power levels, Settings 1, 3, and 5, which corresponded to 50, 90, and 120 W treatments, respectively. Once treatment was completed, the power source was turned off before the sample was removed from under the DBD electrode. For live basil plants treated with CAP, the fine adjustment stand was placed atop a larger adjustable stand to avoid excessive bending of the basil stem to which the treated leaf was attached. Sample Sites 1 (adjacent leaf), 2 (neighboring leaf), and 3 (nearest branched leaf) were also identified before CAP treatment (Figure 1). The directly treated leaf was termed Site 0 (Figure 1). Six biological replicates were used for each treatment.

### 2.3 Rapid quantification of ROS

The fluorescein assay was used to determine the generation of reactive oxygen species (ROS) in basil after CAP treatment. This method was used as the ROS levels in leaves could be measured rapidly after treatment with little additional handling of leaves after separating the four sampling sites. Fluorescein loses its fluorescence upon oxidation by diverse oxidizing species and has been extensively used in an antioxidant assay, oxygen radical absorbance capacity (Gomes et al., 2005).

After sanitization treatment, each of the leaves from each of the sampling sites was weighed and then added to a separate Whirl‐Pak bag (Whirl‐Pak) containing 5 mL of 1 µM fluorescein solution and homogenized in a stomacher (Seward Stomacher 80) on the high setting for 2 min. After homogenization, three 100 µL aliquots per sample were placed in an opaque 96‐well plate and read for fluorescence at an excitation wavelength of 485 nm and an emission wavelength of 525 nm. Fluorescence readings were performed using a Spectramax M5e plate reader (Molecular Devices). Fluorescence values were normalized compared to control (untreated leaves) using the following equation:

(1) $$\mathrm{Normalized}\;\mathrm{fluorescence}=\frac{FL_{s}\times \frac{\left(m_{s}+5\right)}{m_{s}}\times 100}{FL_{c}\times \frac{\left(m_{c}+5\right)}{m_{c}}}$$

where *FL_s_* and *FL_c_* are measured fluorescence values from samples and controls, respectively; *m_s_* and *m_c_* are the leaf mass of sample and control, respectively, and 5 is the volume of fluorescein solution added to the leaf (mL).

### 2.4 Leaf microbiome sequencing

To characterize the basil microbiomes, leaf tissue was processed for 16S rRNA gene sequencing analysis. All samples were frozen at −20°C and lyophilized in a Home Pro Freeze Dryer (Harvest Right, Salt Lake City, UT). Genomic DNA was extracted from approximately 25 mg of leaf tissue using the DNeasy Plant Pro kit (Qiagen, Germantown, MD). Amplicon libraries were prepared in 20 µL PCR reactions with 0.25 µM V3–V4 16S rRNA gene primers from the Quick‐16S Plus NGS Library Prep Kit (Zymo Research, Irvine, CA), 1 U of Phusion high‐fidelity (HF) DNA polymerase (Thermo Scientific, Waltham, MA), 1× Phusion HF reaction buffer, 3% dimethyl sulfoxide, 200 µM deoxynucleoside triphosphates, and 2 µL of template DNA. PNA clamps (1 µM pPNA and 1 µM mPNA; PNA Bio) were included in each reaction to limit amplification of plastid and mitochondrial sequences. PCR reactions were performed on a SimpliAmp Thermal Cycler (Thermo Scientific) with settings for initial denaturation at 95°C for 10 min, followed by 35 cycles at 95°C for 30 s, 78°C for 10 s (annealing of PNA clamps), 55°C for 30 s, and 72°C for 3 min, and final elongation at 72°C for 10 min. PCR products were pooled at equal volumes and cleaned with the Monarch PCR Cleanup Kit (NE Biolabs, Ipswich, MA). DNA concentration of the final pool was quantified with a Qubit 4 Fluorometer (Invitrogen). Paired‐end sequencing (2 × 300 cycles) was performed on an Illumina NextSeq1000 at the Joint Institute for Food Safety and Applied Nutrition (JIFSAN).

Sequenced reads were analyzed using QIIME2 v.2023.5 (Bolyen et al., 2019). Paired‐end sequences were denoised with the DADA2 workflow to generate amplicon sequence variants (ASVs), and taxonomy was assigned using the BLAST method with the SILVA database v.138. Sequences that were classified as mitochondria and chloroplast were removed, and the remaining sequences assigned at the phylum level were included in subsequent analysis. The downstream microbiome analysis was performed in R v.4.3.2. ASV counts were processed for quality control with Source tracking for Contamination Removal in microBiomes (SCRuB) method (version 0.0.1, Austin et al., 2023) to remove potential sequencing contaminants in samples based on negative controls for PCR reagents (*n* = 1) and DNA extractions (*n* = 3). The final ASV table was normalized to relative abundances. Vegan package (Oksanen et al., 2022) was used to determine α‐diversity as Shannon index and β‐diversity via nonmetric multidimensional scaling based on Bray–Curtis dissimilarity.

### 2.5 Statistical analysis

Six live plants were used for control and each treatment. Statistical analysis of fluorescence loss was performed using a paired *t*‐test. Statistical analysis for fluorescein experiments was performed in Excel with an *α* of 0.05. For the microbiome data, permutational multivariate analysis of variance (PERMANOVA) was applied to evaluate effects of CAP (i.e., 50, 90, and 120 W) and leaf sampling distance (0–3). To further determine treatment effects on specific taxa, ASVs present in less than 10% of samples were filtered out, and the Top 20 most abundant bacterial families were evaluated (Nearing et al., 2022). Differential abundances based on CAP intensity and leaf sampling distance were determined using the Kruskal–Wallis test, followed by Dunn's test with false discovery rate correction for multiple comparisons.

## 3 RESULTS

Basil leaves exhibited substantial ROS generation in response to treatment with CAP. Figure 1 shows that normalized fluorescence from fluorescein was measured following incubation with leaves as a function of CAP treatment wattage and distance from the site of CAP application. All treatment sites (i.e., Distances 1–3) at all power levels (i.e., 50, 90, and 120 W) experienced significant increases in ROS concentration in leaf tissue as evidenced by lower fluorescence values normalized with respect to untreated leaves. At Site 0, the actual treatment site, fluorescence was reduced by an average of 84.37% compared to control (no CAP treatment) across all treatment levels, although no significant difference in fluorescence loss was observed between treatment wattage (*p* > 0.5). Although a qualitative trend suggested a slightly lower level of ROS generation in Sites 1–3 compared to Site 0, as evidenced by higher average fluorescence values, there was no significant difference between Sites 1–3 (*p* > 0.05) within a treatment and only an occasional difference with respect to Site 0 (*p* < 0.05). These results show that the similar levels of oxidative stress were experienced not only by the basil leaves that were exposed to CAP treatment but also by leaves that were near the treatment site.

**FIGURE 2:**
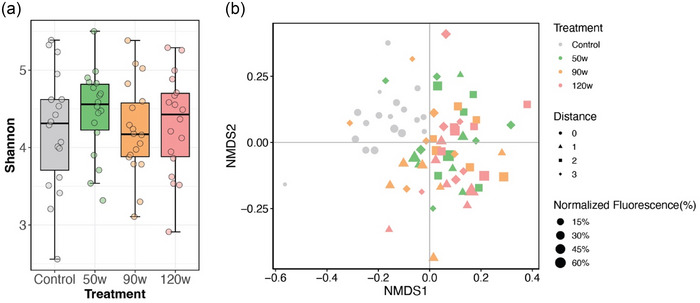
(a) α‐Diversity (Shannon index) of the basil leaf microbiota at the amplicon sequence variant (ASV) level for plants treated with cold plasma. (b) Compositional similarity or β‐diversity of the basil leaf microbiota at the family level for plants treated with cold plasma. Color corresponds to treatment intensity (0 W‐control, 50, 90, and 120 W); shape corresponds to leaf sampling distance (0‐control and following treatment, leaf positions 1–3); size of the shape corresponds to the level of normalized fluorescence (%).

The 16S rRNA gene amplicons for basil plant leaf samples (*n* = 72) and controls (*n* = 5) yielded 39,985,741 paired‐end sequenced reads. Following quality control and preprocessing, there were 46,444 ± 3211 high‐quality assigned reads per leaf microbiome sample (mean ± standard error). CAP treatment did not have significant impacts on α‐diversity of the leaf microbiomes (*p* = 0.60, Figure 2a), indicating that the richness and evenness of ASVs detected were not altered by the treatments. Alternatively, cold plasma appeared to drive major restructuring of the overall microbiota profile (Figure 2b). Microbiomes of plants treated with cold plasma at 50, 90, and 120 W all clustered away from pretreatment controls, and there was a significant effect of treatment (PERMANOVA *p* < 0.05; Figure 2b). Notably, there were no significant differences between the different treatment groups (i.e., 50, 90, and 120 W; PERMANOVA *p* > 0.05 for each pairwise comparison), suggesting that CAP effects on the plant microbiota were variable and not dose dependent. Nevertheless, leaf microbiomes of the treated plants appeared to differ based on leaf sampling distance from plasma application. Bacterial communities of leaves in position 1 (i.e., the closest leaf to CAP exposure) clustered significantly from those associated with leaves in Position 2 or 3 (i.e., further from exposure) (PERMANOVA *p* < 0.05; Figure 2b). Moreover, the fluorescence intensity associated with ROS production significantly affected the bacterial community structure (PERMANOVA *p* < 0.05; Figure 2b). Thus, cold plasma induced significant shifts in the basil leaf microbiota across all treatment intensities, and leaves closest to the plasma application exhibited the most change. Site 0 (treatment site) could not be utilized for 16S rRNA gene sequencing as it was used for fluorescence measurement.

**FIGURE 3:**
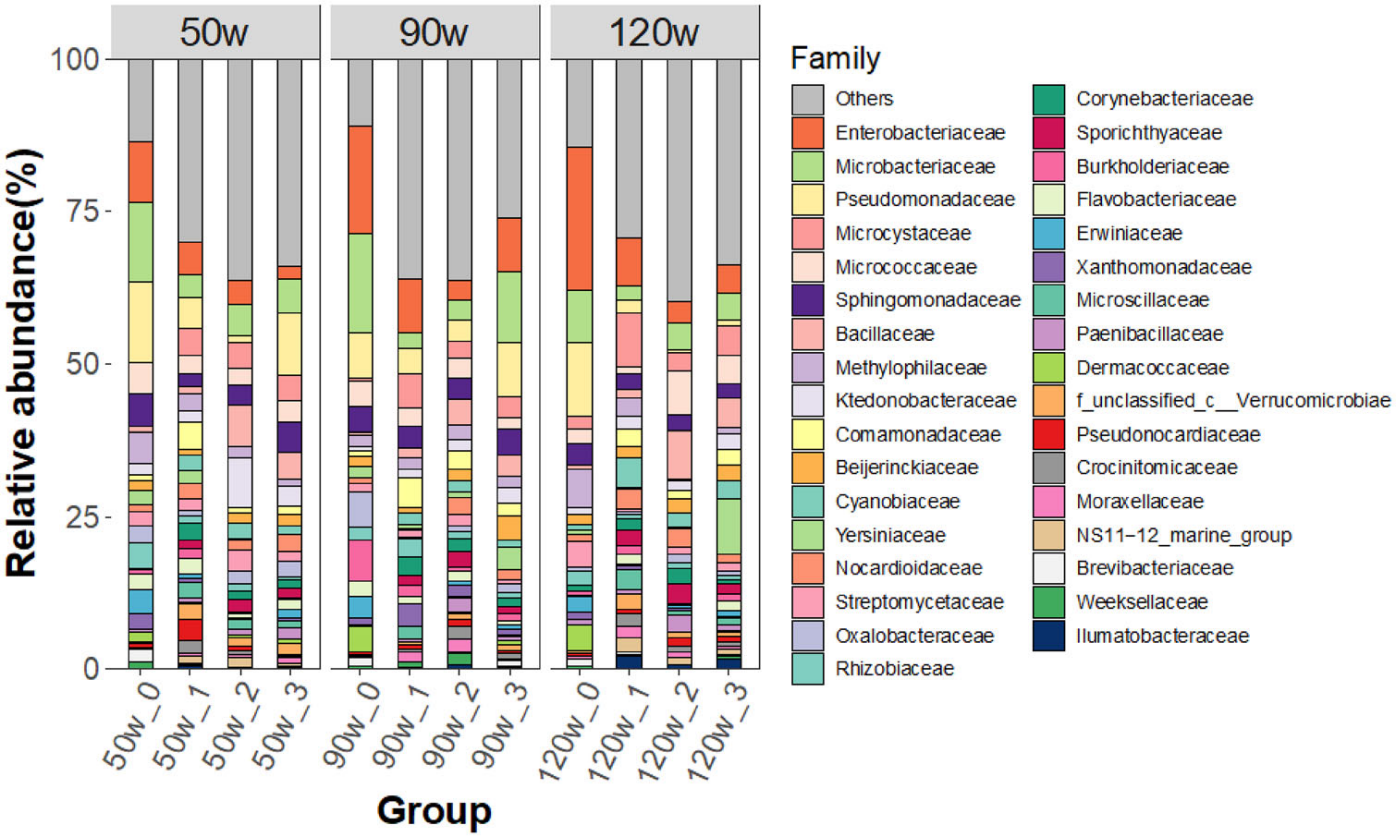
Relative abundances of bacterial families in leaf microbiomes of basil plants treated with cold plasma (50, 90, or 120 W). Within each group, 0 represents the pretreatment control and 1–3 indicate leaf sampling positions following cold plasma treatment. Each bar represents average microbiota profile (*n* = 6 plant replicates). Bacterial families with an average relative abundance >2% across all samples are displayed and the less abundant taxa are grouped as “Others.”

CAP caused suppression and expansion of a variety of dominant bacterial taxa associated with the basil leaves (Figure 3). For example, the relative abundances of Enterobacteriaceae (*p* = 0.051), Microbacteriaceae (*p* = 0.006), Pseudomonadaceae (*p* = 0.003), Streptomycetaceae (*p* = 0.014), Oxalobacteraceae (*p* = 0.038), and Rhizobiaceae (*p* = 0.048) all decreased following treatment. Alternatively, there were significant increases in relative abundances of Microcystaceae (*p* = 0.001), Bacillaceae (*p* = 0.009), Cyanobiaceae (*p* = 0.001), and Sporichthyaceae (*p* < 0.001). In fact, the latter was not detected on leaves of all control group plants and present in 57.4% of CAP‐treated groups (*n* = 31/54 samples were positive), suggesting an increase after initially being below the detection range. Although the level of CAP intensity did not generally select for specific taxa (i.e., comparing 50, 90, and 120 W), the relative abundances of Pseudomonadaceae and Streptomycetaceae differed between plants that had received 120 W compared to 50 W or 90 W of treatment (*p* < 0.05 for each). Thus, cold plasma treatment had extensive effects on key taxa in the basil leaf microbiomes, with similar impacts across all intensities.

Several taxa were differentially abundant along the gradient of leaf distance from cold plasma application. Relative abundances of Bacillaceae and Cyanobiaceae were significantly different at all sampling distance levels (i.e., leaf positions 1–3; *p* < 0.05 for each pairwise comparison). In addition, between leaf positions 1 and 3, there were differential abundances of Microbacteriaceae (*p* = 0.006) and Comamonadaceae (*p* = 0.039). Between leaf positions 1 and 2, there were differential abundances of Burkholderiacae (*p* = 0.003) and Streptomycetaceae (*p* = 0.029), and the latter further differed between Positions 2 and 3 (*p* = 0.021). Although the CAP treatments were associated with increases or decreases in certain taxa, the fluorescence intensity was not significantly correlated with the relative abundance of any top 20 taxa (*p* > 0.05), perhaps reflecting the variation observed under different CAP intensities. Overall, CAP treatments significantly modified basil leaf microbial composition and community structure at all treatment intensities and effects on certain taxa appeared to reflect distance from plasma application.

## 4 DISCUSSION

In natural and agricultural environments, plants interact with diverse microbial assemblages in complex ways that support metabolic processes involved in health and growth of the plant host (Rodriguez et al., 2019; Trivedi et al., 2020). The plant microbiome is influenced by various biotic and abiotic factors, including plant age and immune response, along with field conditions (Adi Wicaksono et al., 2023; Omae & Tsuda, 2022; Sivakumar et al., 2020). CAP appeared to significantly impact the basil leaf microbiome, albeit with considerable variation, which is consistent with previous applied plant microbiome research (Sun et al., 2023; Tamošiūnė, Gelvonauskienė, Haimi, et al., 2020; Tamošiūnė, Gelvonauskienė, Ragauskaitė, et al., 2020). The observed effects may reflect changes in the phyllosphere microhabitats induced by plasma, including increases in oxidative products that impact leaf wetness, plant‐cell signaling, and nutraceutical production. For example, we hypothesize that oxidative products emitted by CAP can modify aliphatic compounds in the leaf cuticle layer that influence surface wettability and may affect attachment or colonization of transient microorganisms (Sivakumar et al., 2020). Decreases in leaf wetness have been previously linked to *Pseudomonas* dynamics (Grinberg et al., 2019), which were observed following CAP exposure in the present study. Moreover, many reactive products generated in high concentrations by DBD plasma electrodes are known plant defenses, such as flavonoids that play a role in protection against UVB and ozone to mitigate damage to macromolecules and organelles (Kreslavski et al., 2021). Stimulating growth and plant sugar production and availability has implications for opportunistic pathogens or spoilage agents, potentially shortening shelf‐life and increasing food safety risk (Sivakumar et al., 2020). Nevertheless, we identified decreases in general members of Enterobacteriaceae and Pseudomonadaceae, that is, bacterial families that contain taxa often associated with such risks.

In addition to reshaping the microbiota, CAP has applications for biofortification. CAP emits UV light, and light treatments are known to affect the nutraceutical content of plants (Cortleven et al., 2022; Roeber et al., 2022; Thoma et al., 2020). Although the role of UV in microbial inactivation due to CAP treatment is uncertain (Nasiru et al., 2021; Niemira, 2012b), it can be useful to increase the phytochemical contents of leafy produce.

CAP also produces ozone. In fact, the DBD electrode was first created for the purpose of ozone generation, and the compound is one of the reactive products produced in CAP generation (Eliasson et al., 1987; Kogelschatz, 2003). Ozone has extensive applications in manufacturing as well as food processing because of its reactivity (Sachadyn‐Król & Agriopoulou, 2020; Sharma et al., 1996; Tamaoki, 2008; Xu et al., 2015). Ozonated water is often used in leafy greens processing as a sanitizer in the washing process (Prado‐Silva et al., 2015). To prevent damage, plants will expel what ozone they can through their stomata, which may modify the resident microflora on the leaf surface (Erickson, 2012). Although the opened stomata are a potential source of pathogen internalization, it remains unseen how the increased plant defense activity to expel the ozone may affect internalized pathogens. Perhaps the nutrient‐scarce and exposed environment of the leaf surface already selects for microorganisms other than enteric pathogens, which often grow more successfully in soil (Vorholt, 2012), and the treatment‐associated increases in oxidative compounds and surface‐available nutrients may strengthen naturally occurring microbial populations, thereby increasing resistance to colonization by human pathogens (Kroupitski et al., 2019; Tsuda et al., 2009).

Limitations in the present study and in others using CAP applications are related to the heterogenous components and, in turn, somewhat variable effects of the plasma. Despite using a well‐characterized plasma device with air as the charge gas, the exact composition of the resulting plasma was unknown. Thus, the variation we observed in ROS production and microbiome diversity across treatments may be attributed to the complex and dynamic nature of plasma species. Understanding the composition and proportion of plasma species in CAP treatment is at the frontier of plasma research.

Our study demonstrates that CAP modulation of the plant microbiome in basil is plant‐wide beyond the site of exposure, perhaps reflecting a systemic oxidative stress response. Further investigation is necessary to characterize the implications of CAP‐induced oxidative stress and microbial community transitions on produce quality. Preharvest CAP treatment may have broad effects on leaf tissue and associated microflora that are not confined to the immediate treatment area because of the plant stress response. More research is needed to understand how the effects of plasma treatment on both the plant stress response and microflora may interact to influence the plants’ susceptibility to human pathogen contamination. Findings from this study open a new avenue for food science and technology researchers to apply CAP technology in the preharvest setting to improve postharvest produce shelf‐life, quality, and safety.

## 5 CONCLUSION

CAP treatment of live basil plant resulted in increased plant stress response in the form of elevated production of ROS, not just at the site of CAP application, but also in the leaves in the vicinity of the treatment site, indicating that this signal propagates through the plant. CAP treatment also caused significant restructuring of the microbial communities of the plant. The leaf microbiota shifts were significant for all CAP intensities and correlated with the distance from the treatment site. Further research is necessary to determine whether elevated ROS production in leaves results in higher production of phenolic compounds. Exploring how the shifts in the leaf microbiota triggered by CAP treatment affect the ability of human pathogenic and spoilage microorganisms to attach, colonize, and survive on the leaf surface has applications for enhancing produce shelf‐life and safety. These results show an emerging direction in CAP research.

## AUTHOR CONTRIBUTIONS

**Andrea R. Gilbert‐Eckman**: Conceptualization; methodology; data curation; investigation; formal analysis; visualization; writing—original draft. **Mairui Gao**: Methodology; investigation; formal analysis; visualization; writing—review and editing. **Ryan A. Blaustein**: Conceptualization; methodology; formal analysis; investigation; visualization; writing—review and editing; supervision. **Rohan V. Tikekar**: Conceptualization; methodology; supervision; funding acquisition; project administration; writing—review and editing; investigation.

## CONFLICT OF INTEREST STATEMENT

The authors declare no conflicts of interest.

## Data Availability

ROS production data are available upon a reasonable request. Raw reads from 16S rRNA sequencing of the basil leaves are available under NCBI BioProject accession PRJNA1182343. Data and scripts that may be used to reproduce the microbiome analysis are available at https://github.com/rablaustein/CAP_basil_microbiome.
